# Supporting Older Adults in Exercising With a Tablet: A Usability Study

**DOI:** 10.2196/11598

**Published:** 2019-02-01

**Authors:** Sumit Mehra, Bart Visser, Nazli Cila, Jantine van den Helder, Raoul HH Engelbert, Peter JM Weijs, Ben JA Kröse

**Affiliations:** 1 Applied Psychology Faculty of Applied Social Sciences and Law Amsterdam University of Applied Sciences Amsterdam Netherlands; 2 CREATE-IT Applied Research Centre Faculty of Digital Media and Creative Industries Amsterdam University of Applied Sciences Amsterdam Netherlands; 3 Informatics Institute Faculty of Science University of Amsterdam Amsterdam Netherlands; 4 Amsterdam Centre for Innovative Health Practice Faculty of Health Amsterdam University of Applied Sciences Amsterdam Netherlands; 5 Faculty of Sports and Nutrition Amsterdam University of Applied Sciences Amsterdam Netherlands; 6 Department of Rehabilitation Amsterdam Movement Sciences Academic Medical Center Amsterdam Netherlands; 7 Department of Internal Medicine, Nutrition and Dietetics VU University Medical Centre Amsterdam Netherlands; 8 Amsterdam Public Health Research Institute Department of Epidemiology and Biostatistics VU University Medical Center Amsterdam Netherlands

**Keywords:** frail elderly, aged, activities of daily living, exercise, health behavior, telemedicine, mobile devices, tablet computers, usability testing, mobile phone

## Abstract

**Background:**

For older adults, physical activity is vital for maintaining their health and ability to live independently. Home-based programs can help them achieve the recommended exercise frequency. An application for a tablet computer was developed to support older adults in following a personal training program. It featured goal setting, tailoring, progress tracking, and remote feedback.

**Objective:**

In line with the Medical Research Council Framework, which prescribes thorough testing before evaluating the efficacy with a randomized controlled trial, the aim of this study was to assess the usability of a tablet-based app that was designed to support older adults in doing exercises at home.

**Methods:**

A total of 15 older adults, age ranging from 69 to 99 years old, participated in a usability study that utilized a mixed-methods approach. In a laboratory setting, novice users were asked to complete a series of tasks while verbalizing their ongoing thoughts. The tasks ranged from looking up information about exercises and executing them to tailoring a weekly exercise schedule. Performance errors and time-on-task were calculated as proxies of effective and efficient usage. Overall satisfaction was assessed with a posttest interview. All responses were analyzed independently by 2 researchers.

**Results:**

The participants spent 13-85 seconds time-on-task. Moreover, 79% (11/14)-100% (14/14) participants completed the basic tasks with either no help or after having received 1 hint. For expert tasks, they needed a few more hints. During the posttest interview, the participants made 3 times more positive remarks about the app than negative remarks.

**Conclusions:**

The app that was developed to support older adults in doing exercises at home is usable by the target audience. First-time users were able to perform basic tasks in an effective and efficient manner. In general, they were satisfied with the app. Tasks that were associated with behavior execution and evaluation were performed with ease. Complex tasks such as tailoring a personal training schedule needed more effort. Learning effects, usefulness, and long-term satisfaction will be investigated through longitudinal follow-up studies.

## Introduction

### Physical Activity Interventions for Older Adults

Physical activity is vital for a healthy life. A sedentary lifestyle is associated with numerous health-related problems such as obesity, diabetes, cardiovascular diseases, various forms of cancer, and depression [1,2]. Furthermore, for older adults, physical activity can prevent or delay the onset of functional impairments and prolong the ability to live independently [3]. Provided by these well-acknowledged health benefits, community-based physical activity programs have spawned across the world [4,5]. A prototypical example of such a program that has been running for over 35 years in the Netherlands is “More Exercise for Seniors” (*Meer Bewegen voor Ouderen*, abbreviated as MBvO in Dutch). Weekly, 400,000 older adults exercise in a group under the guidance of an instructor. Despite the popularity of this program, however, its effects on physical health appear to be insufficient [6]. In particular, studies show a need for higher frequency and longer exercise duration to capitalize on the health benefits of physical activity [7,8].

To achieve the recommended frequency and duration, a home-based exercise program could prove a useful addition to a community-based program such as MBvO. With the convenience of their home, older adults can continue the exercises they have learned during the weekly community classes. A focus-group study showed that the MBvO participants believed additional home exercises would be useful but also had worries about the safety, self-efficacy, and adherence to such an intervention [9].

### Technology Use

Mobile health (mHealth), that is, the use of mobile devices and wireless technology for medical and health practices [10], is increasingly being used to attain health goals, for instance, increasing physical activity, weight loss, stress reduction, or chronic disease management like diabetes. In 2017, over 325,000 health apps were available for the general public through the various app stores [11]. Health professionals, policy makers, and researchers recognize the opportunity to reach a large audience through developing technology-enhanced interventions for various target populations and health outcomes. Increasing physical activity in older adults is one of such intended health outcomes [12-16]. In contrast to popular belief that older adults are not inclined to use technology, the ownership of tablet computers among older adults is growing rapidly [17-19]. The popularity of tablets stems possibly from its usability. Studies show that older adults are able to operate tablets better than personal computers [20,21] or smartphones owing to their large touchscreen [22]. It is not surprising that recent health interventions for older adults choose tablets as the primary mode of delivery [23-27].

### Development of a Tablet-Based Intervention

To increase the physical activity in older adults and capitalize on the potential of mHealth, a technology-enhanced intervention was developed as part of the Motivating Technology for Older Adults’ Behavior (MOTO-B) and VITal Amsterdam elderly IN the city (VITAMIN) projects. The aim of these projects was to develop an mHealth intervention that can be used in conjunction with existing community-based exercise programs. By supporting older adults to perform exercises at home as well, it helps them to achieve the recommended exercise duration and frequency [7,8].

To develop the intervention, the Medical Research Council (MRC) framework was used [28,29]. This framework describes the process of developing, pilot-testing, assessing the effectiveness, and implementing complex health interventions. As part of the development stage, focus groups were conducted with prospective users, and relevant literature was identified, which led to 3 design considerations [9,30]. First, physical activity should be supported by functional exercises that can be executed safely within a home environment. Second, to facilitate behavior change, the intervention should support self-regulation. Third, a blended approach allows the convenience of a home-based exercise program and the ability to tailor the intervention to individual needs to be combined with the effectiveness of rich feedback and social support.

These design considerations were implemented in a tablet-based app called VITAMIN that delivered a home-based exercise program in conjunction with coaching. Key components were goal setting, the ability to tailor the program to individual needs, video demonstration of functional exercises, rating of exercises, and progress tracking and feedback of a personal coach that could remotely monitor performance. See Mehra et al. [30] for a detailed account of how behavior change principles were translated into the blended intervention.

Prior to evaluating the efficacy of the intervention in terms of health outcomes, the feasibility should be assessed. This stage is often overlooked, leading to efficacy studies of interventions that have not matured yet and problems that could have been prevented with sufficient pilot testing [29]. Usability issues are one of the key factors that determine the success of mHealth interventions [31,32]. Usability is defined as the extent that devices can be operated by users to achieve the specified goals with effectiveness, efficiency, and satisfaction in a specified context of use [33]. In line with the feasibility stage of the MRC framework, this study sets out to investigate the usability of the tablet-supported intervention. The aim was to assess whether first-time users could operate the VITAMIN app that was designed to support older adults in doing home-based exercises. First-time users are older adults that have no prior experience of using the app.

## Methods

### Study Design

Zapata et al [32] conducted a systematic review on how the usability of mHealth apps is being evaluated. The majority of the studies use either interviews or questionnaires to investigate usability. These methods rely on self-report of prospective users after having used the device. These methods are suitable to gauge user satisfaction but in lesser degree effectiveness and efficiency. In contrast, other studies investigate the usability by observing users as they try to complete prescribed tasks on the device. This method is a reliable estimate of effectiveness and efficiency but not user satisfaction. Combining various methods to evaluate usability is therefore the recommended approach, although only a few studies do so [32].

This study used mixed methods to investigate the usability of the VITAMIN app. To evaluate effectiveness and efficiency, user performance was recorded and assessed as they executed tasks in a laboratory setting. Satisfaction was evaluated by asking the participants to “think aloud” during the execution of tasks. This is a common technique used in usability studies where users are requested to verbalize their ongoing thoughts as they execute a task [34]. After performing the tasks, participants were interviewed about their overall impression of the app.

### Participants

A total of 15 older adults, 4 men and 11 women, were recruited from local community centers that offer weekly exercise programs. Inclusion criteria were that the participants be at least 55 years old, living independently at home, and taking part in the weekly exercise classes offered by the community center. Exclusion criteria were mental or physical health conditions that could prevent them of operating a tablet, such as the presence of tremors or cataract. Both the inclusion and exclusion criteria match those of a future randomized controlled trial (NTR5888) and the intended implementation of the intervention as an addition to existing community-based exercise programs [35].

### Materials

#### Tablet Application

**Figure 1:**
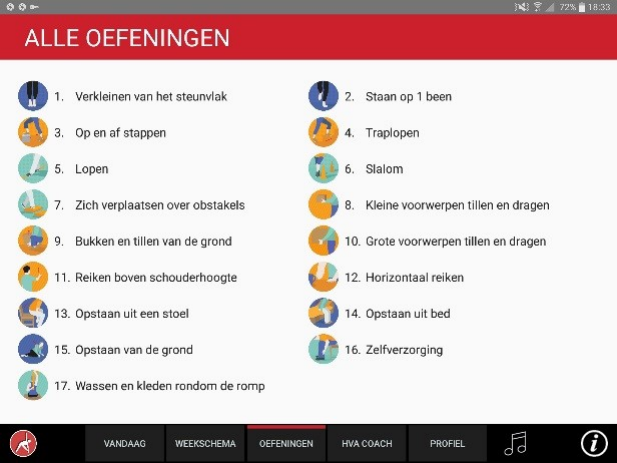
Exercise library.

**Figure 2:**
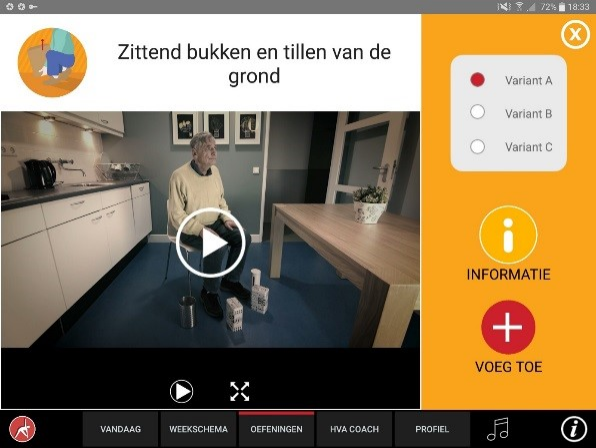
Selecting an exercise variation.

**Figure 3:**
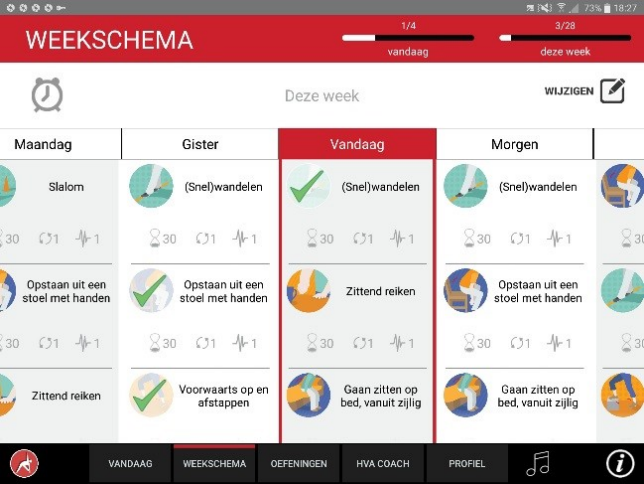
Personal training schedule.

**Figure 4:**
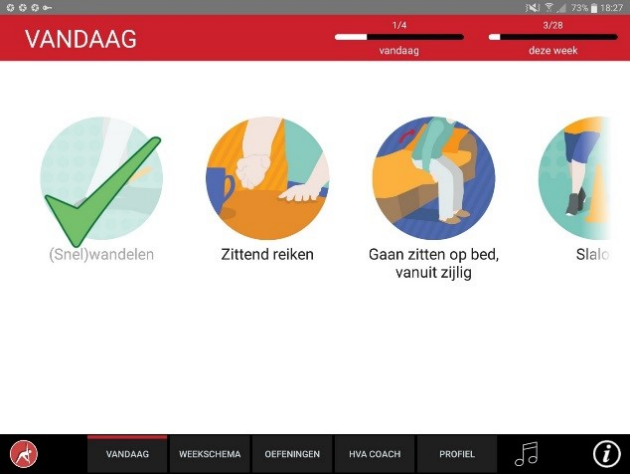
Today’s program.

**Figure 5:**
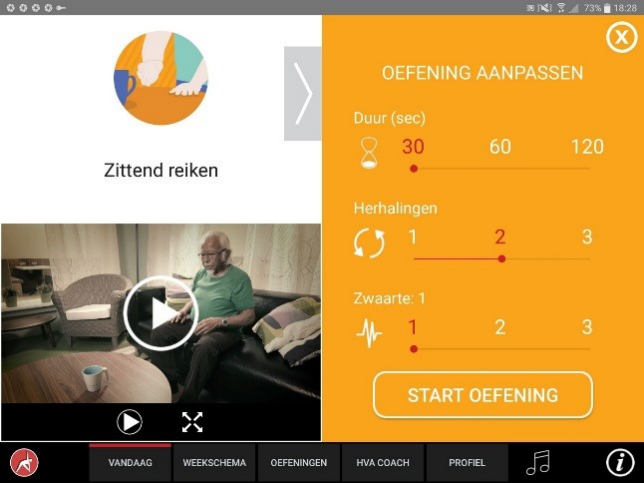
Modifying execution parameters.

**Figure 6:**
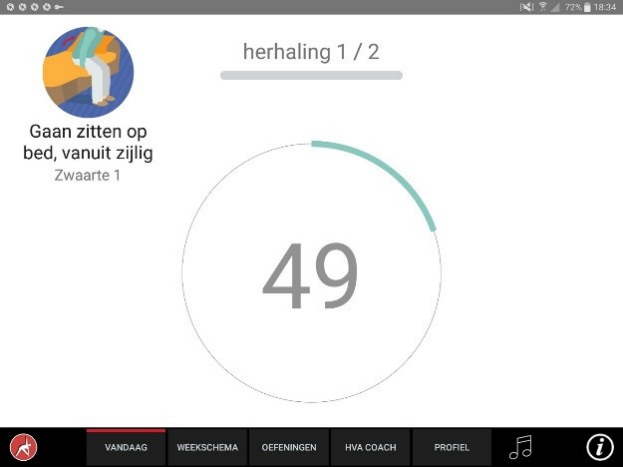
Countdown timer during execution.

**Figure 7:**
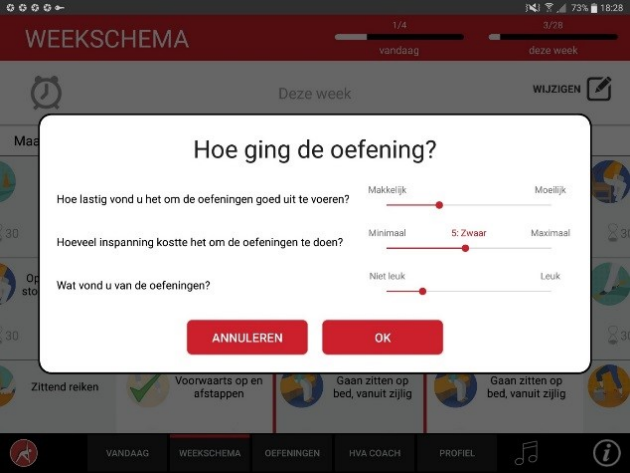
Rating an exercise.

**Figure 8:**
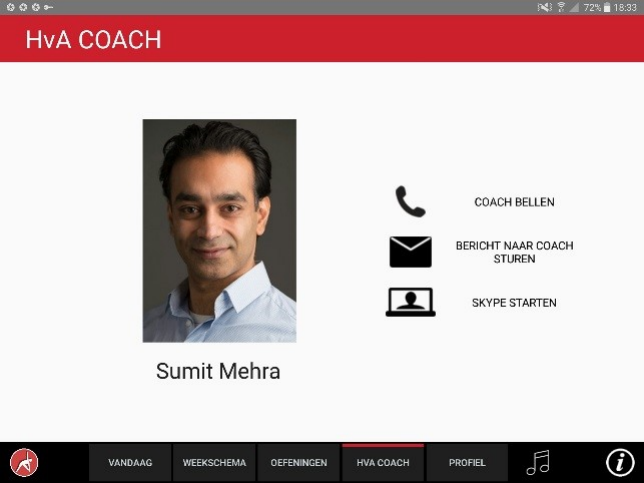
Initiating a video call to the coach.

The app was designed for a 10-inch Android tablet. The main functions of the VITAMIN app were delineated by 5 distinct tabs in the home screen: (1) Exercises, (2) Profile, (3) Weekly Schedule, (4) Today, and (5) Video Calling. *Exercises* is a library that contained 16 functional exercises, designed by human movement scientists, that were devised to be executed in a home setting with ordinary household objects as aids. Each exercise consisted of 3 versions that varied in difficulty. For each variation, a custom-made video with a voiceover was shot (48 in total) that depicted how the exercise could be executed safely (modeling). The video was accompanied by a factsheet that contained background information about the exercise (Figures 1 and 2). *Profile* is the possibility to formulate personal goals and a step-by-step wizard that helped users to set up a weekly schedule with suitable exercises (goal setting & tailoring). *Weekly Schedule* is an overview with icons depicting which exercises were planned for each day of the week (Figure 3). Users could checkmark exercises that had been performed and see, in a glance, what still had to be done (progress tracking). *Today* is a reel of exercises that were planned for that day. To aid the execution, a countdown timer depicted the remaining seconds. Prior to the execution, the user could customize each exercise using 3 parameters: the duration of the exercise, the amount of repetitions of the exercise, and the difficulty level (Figures 4-6). After the completion of each exercise, the user could rate the exercise using 3 scales on difficulty, effort, and fun (Figure 7). *Video Calling* is the option to video call an appointed coach that could motivate and assist the user from distance (motivational interviewing). This coach could also remotely monitor the weekly schedule and the user ratings of each exercise (Figure 8).

The typical use of the app would be exploring the available exercises (1) and setting personal goals (2) during the initial use. The Weekly Schedule (3) and Today (4) tabs are used on a daily basis to assist users in performing their scheduled exercises. Finally, the Video Calling (5) tab is to be used when users want to evaluate and discuss their progress with their personal coach.

#### Usability Tasks

In order to test typical scenarios for novice users that have no to little experience using the app, a series of basic tasks were defined. The tasks were grouped around the 4 tabs: Exercise, Today, Weekly Schedule, and Video Calling described above. The Profile tab could not be tested because it was still in development at the time.

The basic tasks were designed with the novice user in mind. Three additional “expert tasks” were added to the testing procedure as a “back-up option” in case participants completed the basic tasks early. The expert tasks were defined as tasks that would be indicative for advanced users that have been using the app for an extended period of time (see Textbox 1 for a description for the basic and expert tasks that were tested).

**Description of the tasks that were performed by the participants.**

- Today:

Today1: Execute the exercises that are scheduled for today. Adjust the duration to 10 seconds and set the repetition to 1.

Today2: After completing an exercise, rate the difficulty, effort and fun using three scales.

Today3: Find and watch the instructional video of exercise X.

Today4 (expert): During the execution of an exercise, pause the countdown timer.

- Weekly Schedule:

Schedule1: Look up which exercises are planned for Friday.

Schedule2: Add an exercise to your weekly schedule that will increase your capacity to pick up objects from the floor.

Schedule3: In the weekly schedule, remove exercises so that the maximum exercises for that day is three.

Schedule4: Set an alarm so that you will get a daily reminder at 12.00.

Schedule5 (expert): Yesterday you forgot to mark your exercises as completed. Do this in retroaction.

- Exercises:

Exercise1: Look up information about exercise X.

Exercise2 (expert): Study the different variations of exercise X.

- Video Calling:

Video1: Make a video call to your coach.

### Procedure

Participants were received in the usability lab of the university by an experimenter and an assistant. After signing an informed consent document and receiving a short verbal introduction, they were seated behind a desk. The participants were instructed to think aloud as they performed each task. If needed, they were encouraged to do so by asking “what do you see?” or “what are you trying to achieve?” during the experiment. If participants were stuck during the execution of a task, they were given a verbal hint by the experimenter after 30 seconds, for instance “the button you are looking for can be found in the top left-hand corner.” In this manner, the participant could continue with the rest of the task.

After practicing the procedure with a trial run, they were asked to perform the tasks as described in Textbox 1. The order of the tasks was fixed in principle, but some tasks were skipped if the experimenter felt this was appropriate. Occasionally, some participants deviated from the goal and explored the functions of the app. In some cases, this situation made certain future tasks irrelevant. For instance, if a participant already deliberately removed exercises from the weekly schedule during the task Schedule2, performing Schedule3 was skipped for that specific participant. Furthermore, the expert tasks were given only to the participants whose pace was high and when the experimenter believed that the participant would be able to complete all the tasks within the allocated time.

After completing the tasks, the tablet was put aside and the participants were shortly interviewed about their general impression of the app. The sessions lasted 45 minutes in total and were video recorded. Furthermore, the user’s interaction with the tablet was recorded by screen capture software.

### Data Analysis

All recordings were transcribed and coded using software for qualitative analysis (MaxQDA). Two researchers independently coded 4 metrics of the aggregated dataset:

1. Time-on-task: the average time the participants spent on executing a task.
2. Hints: the average number of hints that were given during the execution of a task.
3. Success rate: the proportion of participants that completed the task successfully without any hints, completed the task successfully with hints, and could not complete the task.

Errors: the average amount of errors that were made by participants during the execution of a task. A distinction was made between the following: strategy errors: not knowing how to approach the task (eg, not knowing how to add exercises to the weekly schedule); interaction errors: not knowing how to execute the strategy (eg, unable to find the play button); and operating errors: being unable to operate the device (eg, swiping).

Furthermore, the remarks of the participants during the execution of a task (think-aloud protocol) and posttest interview were classified as either positive, neutral, negative, or a suggestion for improvement.

After both coders annotated the data independently, they compared the results. Differences were resolved via discussion. If no consensus was achieved, the first author settled the rare dispute.

## Results

### Participant Characteristics

The ages of the 15 participants varied from 69 to 99 years old with an average of 77 years (SD 8.5). The majority indicated they had no prior experience operating a tablet.

### Time-on-Task, Success Rate, and Satisfaction of Basic Tasks

The results of 1 participant were excluded from the study because she turned out to be insufficient in Dutch to understand the assigned tasks, and her responses could not be coded reliably. The remaining participants spent 13-85 seconds time-on-task for the basic tasks that were indicative for novice users. Depending on the task, 79% (11-14)-100% (14/14) of the participants completed the tasks successfully with either no help or after having received 1 hint.

Despite the fact that the tasks could be completed successfully by the majority of the participants, their performance varied greatly across different tasks. Executing an exercise (Today1), watching an instructional video (Today3), and video calling a coach (Video1) were conducted relatively easy, as demonstrated by the high success rate without any help. In contrast, adding an exercise to the weekly schedule (Schedule2) appeared to be a more difficult task, indicated by the relatively high failure rate (see Table 1 for the average time-on-task, amounts of hints given, and success rate for the basic tasks). The type of errors that were made ranged from strategy and interaction errors to operating errors (Table 2).

In addition to task performance, the satisfaction per task was assessed with the think-aloud protocol. The majority of the basic tasks elicited more positive remarks than negative remarks during the execution (see Table 3 for the type of remarks per task). Participants were most positive about performing the daily exercises from the Today tab (Today1). This task elicited 3 times more positive remarks than negative remarks. Examples are “I think this is great. A short break. A[n] interval,” “...yes, very easy,” and “...this is very convenient” or “it is quite orderly.” In contrast, the participants were not enthusiastic about looking up information in the Exercise library (Exercise1). During this task, participants could read background information about an exercise. This task elicited 2 times more negative remarks than positive remarks. Examples are “I think this is a lot of text” or “...this is not of much use.” The suggestions made by the participants were “...look, you call it domain. I would use a different term for this” or “I think the text should be shorter.” Also, for watching an instructional video (Today3), participants had several suggestions about enlarging the video to full screen, for example, “enlarging with two fingers would be useful” or “a different symbol for enlarging the video would perhaps be better.”

**Table 1:** Participants who performed the task (N), average time-on-task, number of hints given, and success- and failure rates for basic tasks.

Basic task	Participants, n	Time-on-task (s)	Hints	Success without hints, n (%)	Success with hints, n (%)	Failure, n (%)
Today1	14	78	1.0	10 (71)	3 (21)	1 (7)
Today2	14	59	0.9	6 (43)	8 (57)	0 (0)
Today3	12	20	0.8	7 (58)	4 (33)	1 (8)
Schedule1	12	33	0.8	5 (42)	5 (42)	2 (17)
Schedule2	14	85	0.9	2 (14)	9 (64)	3 (21)
Schedule3	11	60	0.9	0 (0)	10 (91)	1 (9)
Schedule4	13	85	1.1	6 (46)	6 (46)	1 (8)
Exercise1	13	19	0.8	6 (46)	6 (46)	1 (8)
Video1	11	13	1.1	6 (55)	5 (45)	0 (0)

**Table 2:** Participants who performed the task (n) and the average number of errors made for basic tasks.

Basic task	Participants, n	Strategy errors	Interaction errors	Operation errors
Today1	14	0.8	0.2	0.4
Today2	14	0.2	0.0	0.5
Today3	12	0.0	0.2	0.2
Schedule1	12	0.4	0.1	0.0
Schedule2	14	0.5	0.5	0.7
Schedule3	11	0.5	0.5	0.4
Schedule4	13	0.4	0.4	0.1
Exercise1	13	0.8	0.0	0.2
Video1	11	0.1	0.0	0.0

**Table 3:** Participants who performed the task (n) and the total number of remarks evaluated as either positive, negative, neutral, or a suggestion for basic tasks.

Basic task	Participants, n	Positive	Negative	Neutral	Suggestions
Today1	14	18	6	2	1
Today2	14	3	1	3	0
Today3	12	8	4	0	5
Schedule1	12	1	2	1	0
Schedule2	14	3	1	1	1
Schedule3	11	3	1	0	3
Schedule4	13	9	9	0	7
Exercise1	13	4	8	1	8
Video1	11	3	2	0	2

### Time-on-Task, Success Rate, and Satisfaction of Expert Tasks

Besides the basic tasks, a few participants also completed the expert tasks. The time-on-task varied from 14 to 58 seconds. The success rate varied from 75% to 100%. As could be expected, more hints were needed to complete the tasks successfully compared with the basic tasks described earlier. Marking an exercise retroactively as completed, which required the participant to tap and hold down for a certain amount of time, proved to be an especially difficult task. This task had the highest time-on-task, failure rate, and errors. The verbal remarks of the participants indicated that they appreciated the possibility of retroactively marking exercises as complete but found its operation difficult (see Tables 4 and 5 for details of the expert task performance; see Table 6 for the type of remarks per task).

**Table 4:** Participants who performed the task (n), average time-on-task, number of hints given, and success and failure rates for expert tasks.

Expert task	Participants, n	Time-on-task (s)	Hints	Success without hints, n (%)	Success with hints, n (%)	Failure, n (%)
Today4	4	14	3.5	2 (50)	1 (25)	1 (25)
Schedule5	8	58	1.5	1 (13)	5 (63)	2 (25)
Exercise2	4	18	3.5	1 (25)	3 (75)	0 (0)

**Table 5:** Participants who performed the task (n) and average number of errors made for basic tasks.

Expert task	Participants, n	Strategy errors	Interaction errors	Operation errors
Today4	4	0.0	0.5	0.0
Schedule5	8	0.4	0.4	0.6
Exercise2	4	0.8	0.0	0.0

**Table 6:** Participants who performed the task (n) and the total number of remarks evaluated as either positive, negative, neutral, or a suggestion for expert tasks.

Expert task	Participants, n	Positive	Negative	Neutral	Suggestions
Today4 (expert)	4	5	1	0	0
Schedule5 (expert)	8	3	2	1	2
Exercise2 (expert)	4	1	1	0	1

### Overall Satisfaction

During the posttest interview, the participants were overall positive; 31 positive remarks were made against 10 negative remarks. The number of participants in the posttest interview (n) was 14. In this interview, 31 remarks were validated as positive, 10 as negative, 10 as neutral, and 22 as suggestions. Typical positive remarks were “Nice. I found easy to operate and fun,” “it was pretty clear and straightforward,” and “it’s nice to do different exercises now and then.” Examples of negative remarks were “I am not sure if I would use this app, because it seems to me as an invasion of privacy if every time you have to enter what you have done” or “it wasn’t always clear.” The participants also made several suggestions, often in the line of giving more extensive instructions prior to the first use. A typical remark was “maybe you could provide some more information. Like it works so and so. Perhaps a manual or something.” This bore relevance to the brief verbal introduction they received about the app.

## Discussion

### Principal Findings

Overall, the app that was designed to support older adults in doing exercises at home appears to be usable for first-time users. After a brief introduction, the vast majority of the participants could complete the assigned tasks. They did this not only effectively (as indicated by the high success rate) but also efficiently. Mostly within 1-2 minutes, they successfully performed the tasks. Furthermore, the think-aloud remarks and posttest interview revealed that the users were satisfied with the app in general.

The performance varied from task to task. Basic tasks that were associated with supporting behavior execution (Today and Exercise) and evaluation (Video Calling) were completed successfully by the majority of the participants, whereas tasks that were associated with tailoring (Weekly Schedule) were more difficult for the users, as indicated by the longer task completion times and higher rate of errors.

The fact that the older adults in this usability study needed some minor help with performing the assigned tasks is not considered to be a major issue by the authors. First of all, the average age of the participants was 77 years old. The majority had never operated a tablet before and only received a short introduction of a few minutes before they had to perform the assigned tasks under the scrutiny of 2 observers. Observer effects and the think-aloud protocol are known to decrease performance for complex tasks in usability studies [36-38]. It is plausible that the participants would have performed better in the privacy of their own home where they feel more free from prying eyes. Second, the expert tasks were developed with an experienced “power user” in mind. It was designed in an unobtrusive manner not to clutter the interface for first-time users. Therefore, it was not surprising that the participants in the study, as first-time users, had more difficulties executing those tasks. Third, the app is designed to be implemented in a blended intervention in which a coach will be appointed. This coach will give hands-on support, face-to-face and remotely. Thus, in this particular case, receiving help to operate the app is not an artefact of the usability study but reflects the actual context of use.

### Limitations and Future Work

The app is part of a blended intervention in which older adults participate in weekly group-based classes, perform tablet-supported exercises at home, and receive feedback by a personal coach. This study only evaluates if the app that is part of the blended intervention is usable for older adults. It does not evaluate other aspects of the intervention. Furthermore, the usability study was conducted in a lab where users interacted with the app for a short period of time. It provides an indication of the usability for first-time users but not for long-term users. Learnability and user acceptance can only properly be studied when older adults have used the app for an extensive period of time. To investigate these matters, follow-up studies are planned. A randomized controlled trial will evaluate the efficacy of the blended intervention in terms of health outcomes [35]. Parallel to this randomized controlled trial, participants that have been using the app for 6 to 12 months will be questioned about the perceived usefulness, ease of use, learnability, and satisfaction on the long term [39]. To optimize reliability and validity, both questionnaires and interviews will be used.

### Conclusion

In line with the MRC framework, an evidence-based blended intervention was developed to support older adults in performing functional exercises at home. The feasibility of the tablet-based app that was designed for this purpose has been validated by a usability study with mixed methods. Older adults were able to use the app in an effective and efficient manner. They were mostly also satisfied with the app. These findings pave the way to implement and evaluate the intervention in practice.

## Abbreviations

MBvO: Meer Bewegen voor Ouderen (Dutch for More Exercise for Seniors)
mHealth: mobile health
MOTO-B: Motivating Technology for Older Adults’ Behavior
MRC: Medical Research Council
VITAMIN: VITal Amsterdam elderly IN the city
